# Supplementary material for: Patient Safety Incidents in Primary Care: Comparing APEAS–2007 (Spanish Patient Safety Adverse Events Study in Primary Care) with Data from a Health Area in Catalonia (Spain) in 2019
Source: Healthcare (Basel). 2024 May 25;12(11):1086. doi: 10.3390/healthcare12111086 (PMC11172342; doi:10.3390/healthcare12111086)
Supplement: Supplementary file 1 [file healthcare-12-01086-s001.zip › healthcare-2956751-supplementary.pdf]

Form for the identification of incidents and AE in Primary Care

DATA OF THE CENTER

Select the centre

☐xxxx

DATA OF THE NOTIFIER

Professional category:

☐doctor ☐paediatrician ☐gynaecologis

☐resident doctor

☐nurse ☐midwife ☐Health technician

☐Social worker

☐Administrative*

☐Administrative assistant*

☐Caretaker*

☐Nursing technician*

Work experience:

☐Less than 1 year

☐From 1 to 5 years

☐From 5 to 10 years

☐More than 10 years

* Professionals in these categories must proceed to question n. 3 of the questionnaire

INCIDENT RELATED TO:

☐Assistance

☐Environment of care (no patient directly related to the incident)

PATIENT DATA

Administrative data

Identification:

Age:

Gender: ☐man ☐woman

Date of notification:

Date of event:

Clinical data

1. **Patient characteristics**

   ☐PCC ☐MACA ☐FRAGILE ☐GMA 1 ☐GMA 2 ☐GMA 3 ☐GMA 4 ☐GMA 5

2. **Indicate if the patient has any of the following risk factors**

   INTRINSIC Factor

   ☐Alcoholism ☐Hypoalbuminemia ☐Pressure ulcer

   ☐Liver cirrhosis ☐Immunodeficiency ☐Neoplasia

   ☐Depression ☐Heart failure ☐ Neutropenia

   ☐Diabetes ☐Renal failure ☐Obesity

   ☐Drug addiction ☐Coronary artery disease ☐HIV

   ☐Hypercholesterolemia ☐Chronic lung disease ☐Others: ___________________

   ☐Hypertension ☐Malformations

   ____

Attention: Any printing of this document is considered an uncontrolled copy. Only the document published in the document manager of the ICS Camp de Tarragona is considered controlled.

EXTRINSEC Factor

☐Colostomy ☐Open urinary catheter ☐Tracheotomy

☐Enteral nutrition ☐Closed urinary catheter

☐Other:_________________

☐Nasogastric tube ☐Immunosuppressive therapy _________________________

DESCRIPTION OF THE INCIDENT

3. **Summarize what happened and what caused it:**___________________________

____

____

______

4. **At what level of care did the problem occur?**
   (Tick only one option)
   ☐Medical transport
   ☐Community care
   ☐Urgent care in Primary Care
   ☐ASSIR queries
   ☐Consultations of specialized care
   ☐Primary care nursing consultations
   ☐Primary Care medical consultations
   ☐Home address
   ☐General spaces
   ☐Previous hospital admission
   ☐Laboratory
   ☐Pharmacy warehouse
   ☐Material warehouse
   ☐Community pharmacy office
   ☐Residence
   ☐Nursing technical room
   ☐Citizen attention desk
   ☐Hospital emergencies
   ☐Administrative area
   ☐Others: ____

5. **Type of visit**
   ☐Attended
   ☐Not in person
   ☐Home address

RISK MATRIX

6. **Likelihood of happening**
   (Tick only one option)
   ☐Frequent (It is expected to happen again in the coming weeks or months)
   ☐Probable (Can happen under various circumstances, several times a year)
   ☐Possible/Occasional (May reoccur or occur once or twice a year)
   ☐Infrequent (Could happen sometime every 2 to 5 years)

Attention: Any printing of this document is considered an uncontrolled copy. Only the document published in the document manager of the ICS Camp de Tarragona is considered controlled.

☐Very infrequent (Unlikely to recur – may occur only in exceptional circumstances, more than 5 years)

7. Which of these statements best describes the impact on the patient?
(Tick only one option)
If you check the option "notifiable circumstance, near incident or incident without damage", answer question 8 and go to question 14

☐**Incident that does not reach the patient:**
- ☐Reportable circumstance (effect has not happened, but was about to happen)
- ☐Near miss (the incident has occurred, but was detected before it affected a patient)

☐**Incident that reaches the patient without harm:**
- ☐Non-harm incident (the incident happened and affected a patient, but the patient was not harmed)

☐**Incident that reaches the patient and causes harm (Adverse Event):**
- ☐The effect has passed and the patient required observation
- ☐The effect has worn off and the patient has taken temporary damage
- ☐The effect has passed and the patient has been in a critical situation (Ex.: cardiac arrest)
- ☐The effect has worn off and the patient has suffered permanent damage
- ☐The effect has passed and the result has been the death of the patient

Attention: Any printing of this document is considered an uncontrolled copy. Only the document published in the document manager of the ICS Camp de Tarragona is considered controlled.

CONTRIBUTING FACTORS
**8. Contributing factors related to:**<br>☐**Patient-related factors**<br>☐Distraction or lack of attention<br>☐Failure to comply with rules / orders / instructions given by the professional<br>☐Escape or disappearance<br>☐Misinterpretation of information<br>☐Clinical complexity / Associated diseases<br>☐Communication problems<br>☐Existence of emotional factors<br>☐Negative attitude / Absence of collaboration<br>☐Risk behaviour<br>☐Aggression / Hostility<br>☐Self-harm / autolysis (suicide: attempt or consummation)<br>☐Emergency care situation / Severity<br>☐Transfer / Displacement / Transitions...<br>☐Sensory limitations<br>☐**Factors related to the professional**<br>☐Distraction or lack of attention<br>☐Fatigue or exhaustion<br>☐Failure to comply with rules / orders / directions<br>☐Technical error: slip, oversight or error<br>☐Incorrect selection of available alternatives<br>☐Communication on paper<br>☐Verbal communication<br>☐Excessive attention to an item / action / circumstance<br>☐Overconfidence<br>☐Lack of specific skills<br>☐Ignorance of specific information<br>☐Incorrect application of rules / protocols<br>☐Misinterpretation of information<br>☐Electronic communication<br>☐Communication problems with another professional<br>☐Existence of emotional factors<br>☐Existence of social factors<br>☐**Factors of the physical environment**<br>☐Infrastructures<br>☐Remote or very distant from the service<br>☐Environmental risk assessment or safety assessment<br>☐Current code, specifications or regulations<br>☐Equipment<br>☐**Service organization factors**<br>☐Protocols, guidelines, policies, procedures or processes<br>☐Organizational decisions or culture<br>☐Organization of teams<br>☐Resources or workload<br>☐Availability of operating rooms, beds for income or others<br>☐Staff availability<br>☐Organization of teams

Attention: Any printing of this document is considered an uncontrolled copy. Only the document published in the document manager of the ICS Camp de Tarragona is considered controlled.

☐Access to updated protocols, procedures or circuits

☐**External factors**

☐Natural environment

☐Products, technology and infrastructure

☐Services, systems and policies

CAUSAL FACTORS OF THE ADVERSE EVENT

9. Indicate all causal factors of the adverse event (Risk Map)

Related to medication

☐Adverse drug reaction

☐Medication errors

☐Incorrect dosage

☐Incorrect treatment duration

☐Dispensing error (Community Pharmacy Office)

☐Preparation or manipulation error (Different route of administration)

☐Lack of adherence to treatment

☐Incorrect frequency of administration

☐Drug interaction

☐Expired medication

☐Wrong medication

☐Insufficient monitoring

☐Patient denial

☐Omission of dose, medication or vaccine

☐Wrong patient

☐Prescription NOT effective

☐vaccines

☐Stock and order management

☐Reception

☐Conservation and storage

☐Preparation

☐Administration CAP

☐School administration

☐Calculation problems

☐Registration issues (e-CAP)

☐Expired vaccines

☐**Related to management**

☐Erroneous quote

☐Duplication of the clinical history

☐Mistake in health information

☐Patient identification error

☐Long waiting list

☐Loss of documents

☐Problems with computerized history

☐**Related to diagnosis**

☐Error in diagnosis

☐Delay in referral to specialist care

☐Delay in diagnosis

☐**Related to communication**

Attention: Any printing of this document is considered an uncontrolled copy. Only the document published in the document manager of the ICS Camp de Tarragona is considered controlled.

☐Administrative-Administrative Communication
☐Administrative Communication-nursing technician
☐Administrative-Patient Communication
☐Nursing-Administrative Communication
☐Nurse-Nurse Communication
☐Nurse Communication- nursing technician
☐Nurse-Patient Communication
☐Doctor-Administrative Communication
☐Doctor- nursing technician communication
☐Doctor-Nurse Communication
☐Doctor-Doctor Communication
☐Doctor-Patient Communication
☐Communication with other devices
☐Cultural barrier
☐Language barrier

☐**Related to patient care**

☐Inadequate patient handling
☐Inadequate handling of warning signs
☐Inadequate handling of the technique
☐Inadequate handling of the procedure
☐Inadequate maintenance of catheters

☐**others**

☐Other causes: ____

EFFECTS OF THE ADVERSE INCIDENT

10.Indicate all the effects that occurred in the patient:

☐**Related to a procedure**

☐Hemorrhage or hematoma related to surgery or procedure
☐Hematuria related to probing
☐Circulatory disorder (tight splint)
☐Dehiscence of sutures
☐Serous, abscesses or granulomas
☐Tympanic perforation
☐Other complications due to a procedure

☐**Related to Healthcare Associated Infections (HAIs)**

☐Surgical and/or traumatic wound infection
☐UTI associated with probing
☐Device-associated bacteraemia
☐Opportunistic infection due to immunosuppressive treatment or antibiotic use
☐Pressure sore infection
☐Aspiration pneumonia

☐**Related to patient care**

☐Phlebitis
☐Pressure ulcer
☐Burns, erosions, falls and bruises (including consequential fractures)
☐Sciatica injury from injectable
☐Other consequences of patient care

Attention: Any printing of this document is considered an uncontrolled copy. Only the document published in the document manager of the ICS Camp de Tarragona is considered controlled.

☐**Generals**

- ☐By evolutionary course of the underlying disease
- ☐Need to repeat the procedure or visit
- ☐Anxiety, stress or depression

☐**Related to medication**

- ☐Nausea, vomiting or diarrhoea secondary to medication
- ☐Pain or discomfort due to drugs (epigastralgia)
- ☐Itching, rash or skin lesions reactive to drugs or dressings
- ☐Systematic allergic manifestations
- ☐Drug headache
- ☐Neurological changes due to drugs
- ☐Constipation
- ☐Other side effects (cough, dyspnoea, dry mouth,...)
- ☐Hypotension due to drugs
- ☐Poor blood pressure control
- ☐Upper digestive bleeding
- ☐Haemorrhage due to anticoagulation
- ☐AMI, stroke, PE, DVT
- ☐Electrolyte imbalance
- ☐Edema, heart failure and shock
- ☐Alteration of heart rhythm or electrical activity due to drugs
- ☐Functional alteration (kidney, liver, thyroid,...)
- ☐Poor glycaemic control
- ☐Neutropenia
- ☐Local effects or fever after vaccine or drug
- ☐Poor pain management

☐**Others**

- ☐Other consequences ____

☐**No effect**

CONSEQUENCE OF THE ADVERSE EVENT

11.What care did the patient receive as a result of the adverse event?

- ☐Health care was not affected
- ☐It required a higher level of observation and monitoring in primary care
- ☐Required an additional test (x-ray, analyses,...) in primary care
- ☐Additional medical or surgical treatment (antibiotics, minor surgery,...) in primary care
- ☐Required a new consultation or referral to Specialized Care or Emergency without admission
- ☐Hospitalization required: life support treatment (orotraqueal intubation, CPR, surgical intervention)

12. To what extent has healthcare been the cause of the injury?
(Tick only one option)

- ☐Absent evidence that the incident is due to patient handling, the injury is entirely due to the patient's pathology
- ☐Minimal likelihood that handling was the cause
- ☐Slight chance that handling was the cause
- ☐Moderate probability that handling was the cause
- ☐Handling was most likely the cause

Attention: Any printing of this document is considered an uncontrolled copy. Only the document published in the document manager of the ICS Camp de Tarragona is considered controlled.

☐Full evidence that the handling was the cause of the incident/adverse event

AVOIDABILITY
**13.In your opinion, is there any evidence that AE could have been prevented?**<br>☐Yes ☐No ☐Doubtful<br><br>**14.Evaluate the evidence of the possibility of prevention**<br>**(Tick only one option)**<br>☐Minimal possibility of prevention<br>☐Slight possibility of prevention<br>☐Moderate possibility of prevention<br>☐High possibility of prevention<br>☐Total evidence of the possibility of prevention

SUGGESTIONS FOR IMPROVEMENT
**15.What could have been done to avoid this problem?**<br>____<br>____<br>____<br>_________________________

RESOLUTION
**16.Who decides?**<br>☐Primary care team<br>☐Unit of Patient Safety

TO BE COMPLETED BY THE UFSP
**17.Type of incidents (MADS classification)**<br>☐Urgent care<br>☐Continuity of care<br>☐Education for health<br>☐Ethics and citizens' rights<br>☐Management of clinical material<br>☐Waste Management<br>☐Laboratory<br>☐Care process<br>☐Administrative processes<br>☐Diagnostic Image Service<br>☐General services (cleaning, infrastructure, security and techno-assistance devices)<br>☐Social work<br>☐Safer drug use<br>☐Vaccines<br>☐Surveillance, prevention and infection control<br><br>**18. Quality of notification**<br>**(Tick only one option)**

Attention: Any printing of this document is considered an uncontrolled copy. Only the document published in the document manager of the ICS Camp de Tarragona is considered controlled.

☐Correct
☐Disagreement

19. Who resolves the notification?

☐Primary care team
☐Assistance direction
☐Pharmacy
☐Laboratory
☐Responsible citizenship
☐Transverse Unit of Patient Safety
☐Others

20. Safe practices and/or Unit of Patient Safety improvement actions

☐Improving teamwork_new
☐ Improving teamwork_existing
☐Training action (courses, workshop,...)
☐Informative action (clinical session, mail,...)
☐Commission/committee
☐Review documents
☐Analysis London

21.Safe practices and/or primary care team improvement actions

☐ Improving teamwork_new
☐ Improving teamwork_existing
☐Training action (courses, workshop,...)
☐Informative action (clinical session, mail,...)
☐Committee/management
☐Review documents
☐Analysis London

22.status

☐Solved
☐Pending to solve territorial UFSP
☐Pending to solve DAP
☐Pending to fix other units

Attention: Any printing of this document is considered an uncontrolled copy. Only the document published in the document manager of the ICS Camp de Tarragona is considered controlled.
